# Supplementary material for: A KCa 2.2/2.3 Opener Reverses ET-1-Induced NLRP3 Activation in Hypertensive Mice Corpora Cavernosa
Source: Biomolecules. 2026 Mar 13;16(3):432. doi: 10.3390/biom16030432 (PMC13023931; doi:10.3390/biom16030432)
Supplement: Supplementary file 1 [file biomolecules-16-00432-s001.zip › biomolecules-4187894-supplementary.pdf]

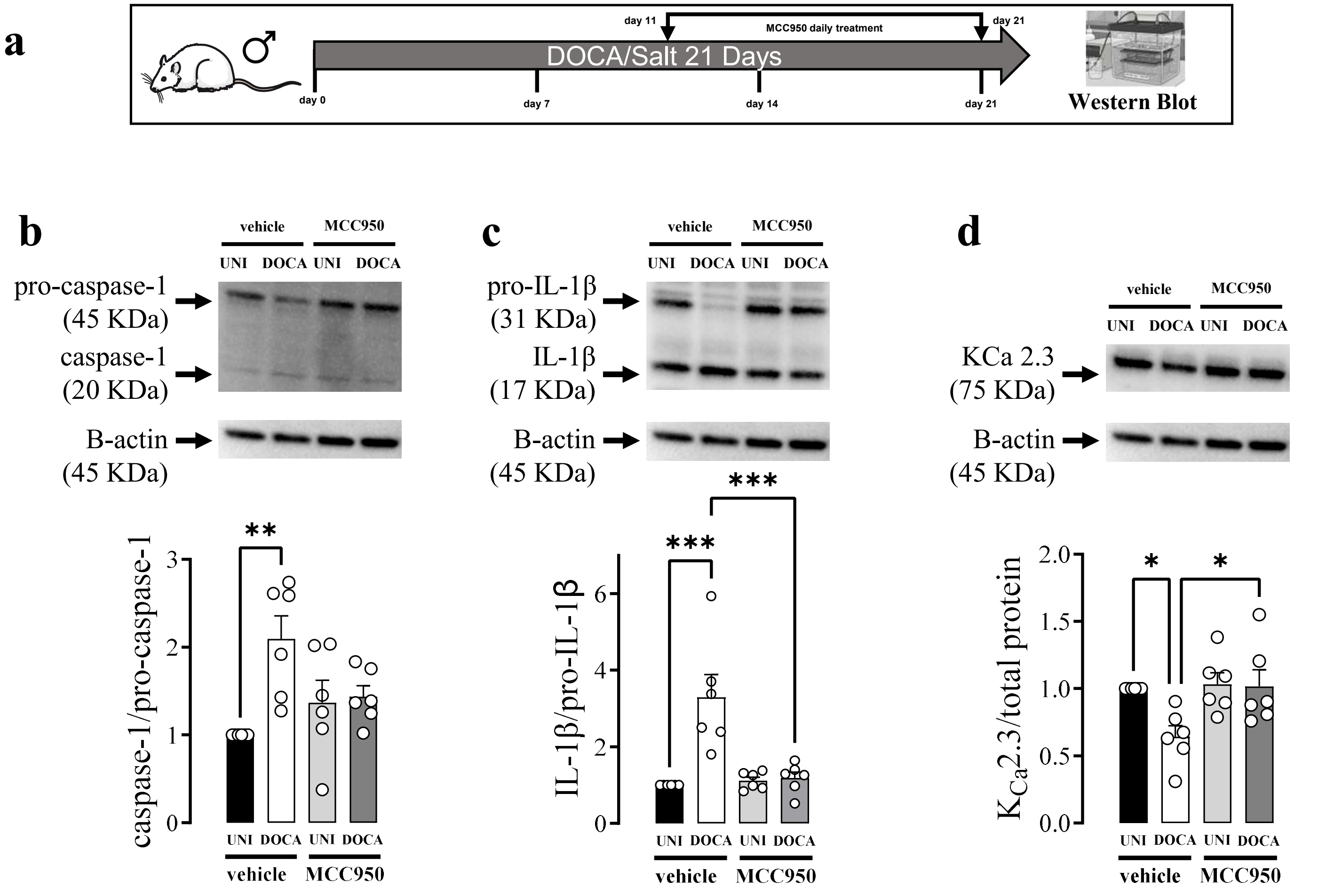

Figure S1. NLRP3 inhibition prevents inflammation in DOCA/salt mice. (**a**) Experimental design. Immunoblot analysis of (**b**) caspase-1, (**c**) IL-1β, and **(d)** $K_{Ca}2.3$ expression in CC strips of UNI and DOCA hypertensive mice treated with MCC950 or vehicle. The arrow indicates when the treatment started (c). Data represent the mean ± SEM values of the groups. * $p<0.05$. $n=6$. The comparison of each value was performed by two-way ANOVA followed by the Holm-Šídák post-test. The entire membranes for pro-caspase-1, caspase-1, pro-IL-1β, IL-1β, and $K_{Ca}2.3$ are shown in figure S12&14.

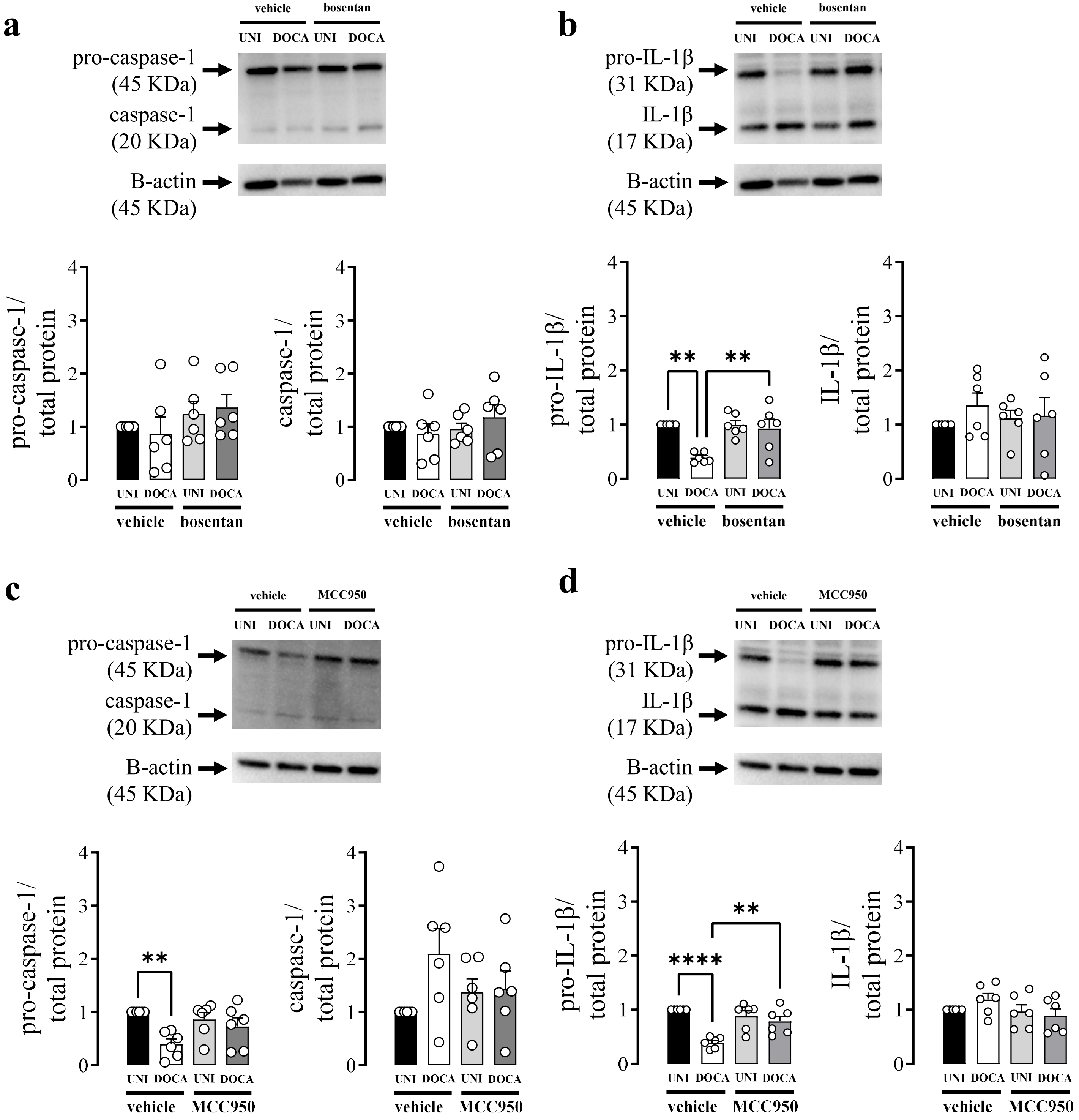

Figure S2. Analysis of the (**a** and **c)** pro-caspase-1and caspase-1, (**b** and **d)** pro-IL-1β and IL-1β expression in CC strips of DOCA/salt hypertensive (DOCA) or unilaterally nephrectomized (UNI) rats treated with vehicle, (**a** and **b**) bosentan or (**c** and **d**) MCC950. Data represent the mean ± SEM values of the groups. * p<0.05. n=6. Each value was compared by two-way ANOVA followed by The Holm-Šídák post-test. The entire membranes for pro-caspase-1, caspase-1, pro-IL-1β, IL-1β, and $K_{Ca}2.3$ are shown in figure S9, S11, S12, and S14.

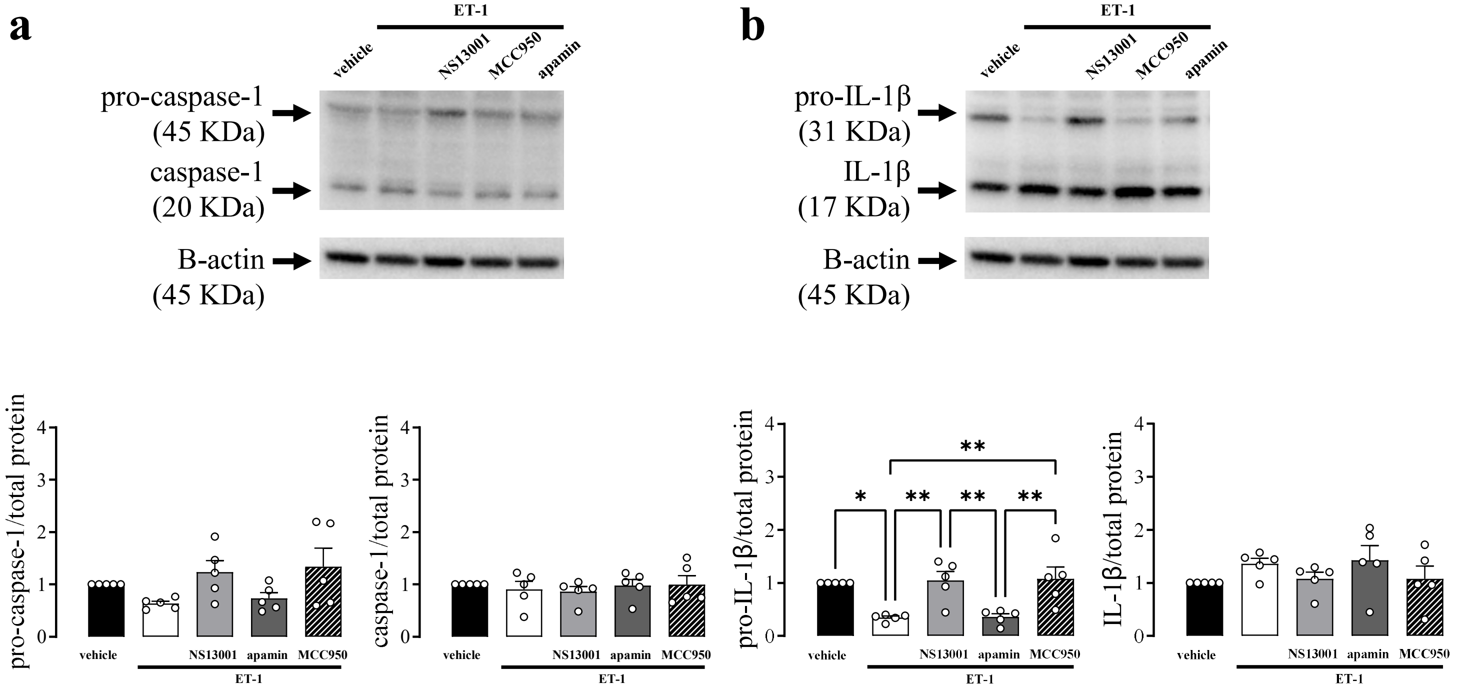

Figure S3. Analysis of the (**a**) pro-caspase-1and caspase-1, (**b**) pro-IL-1β and IL-1β expression in CC strips of healthy mice treated with vehicle, or ET-1 in the presence or absence of NS13001, apamin, and MCC950. Data represent the mean ± SEM values of the groups. * $p<0.05$. n=6. Each value was compared by two-way ANOVA followed by The Holm-Šídák post-test. The entire membranes for pro-caspase-1, caspase-1, pro-IL-1β, IL-1β, and $K_{Ca}2.3$ are shown in figure S18&20.

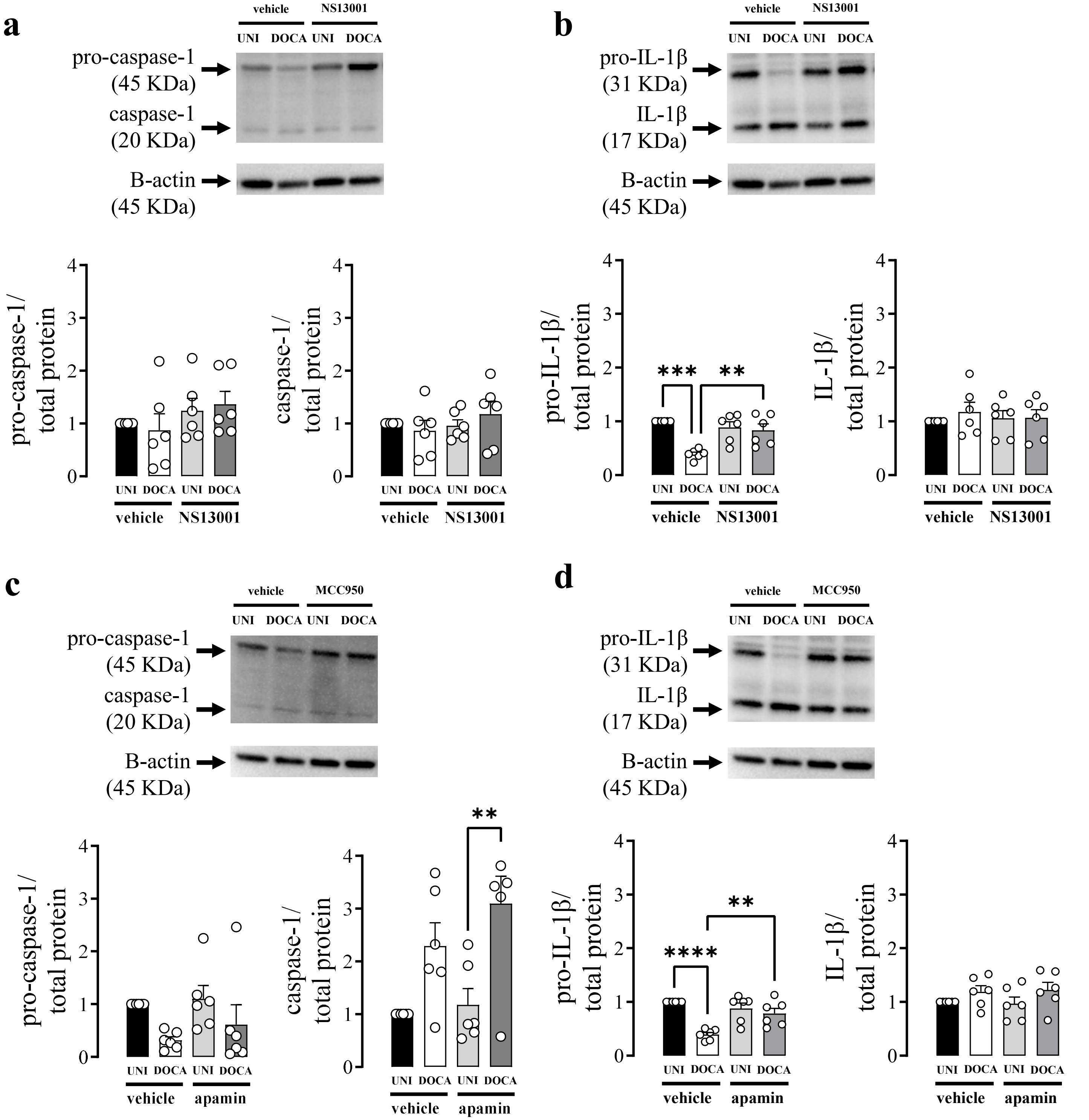

Figure S4. Analysis of the (**a** and **c)** pro-caspase-1and caspase-1, (**b** and **d)** pro-IL-1β and IL-1β expression in CC strips of DOCA/salt hypertensive (DOCA) or unilaterally nephrectomized (UNI) mice treated with vehicle, (**a** and **b**) NS13001 or (**c** and **d**) apamin. Data represent the mean ± SEM values of the groups. * p<0.05. n=6. Each value was compared by two-way ANOVA followed by The Holm-Šídák post-test. The entire membranes for pro-caspase-1, caspase-1, pro-IL-1β, IL-1β, and $K_{Ca}2.3$ are shown in figure S12, S14, S15, and S17.

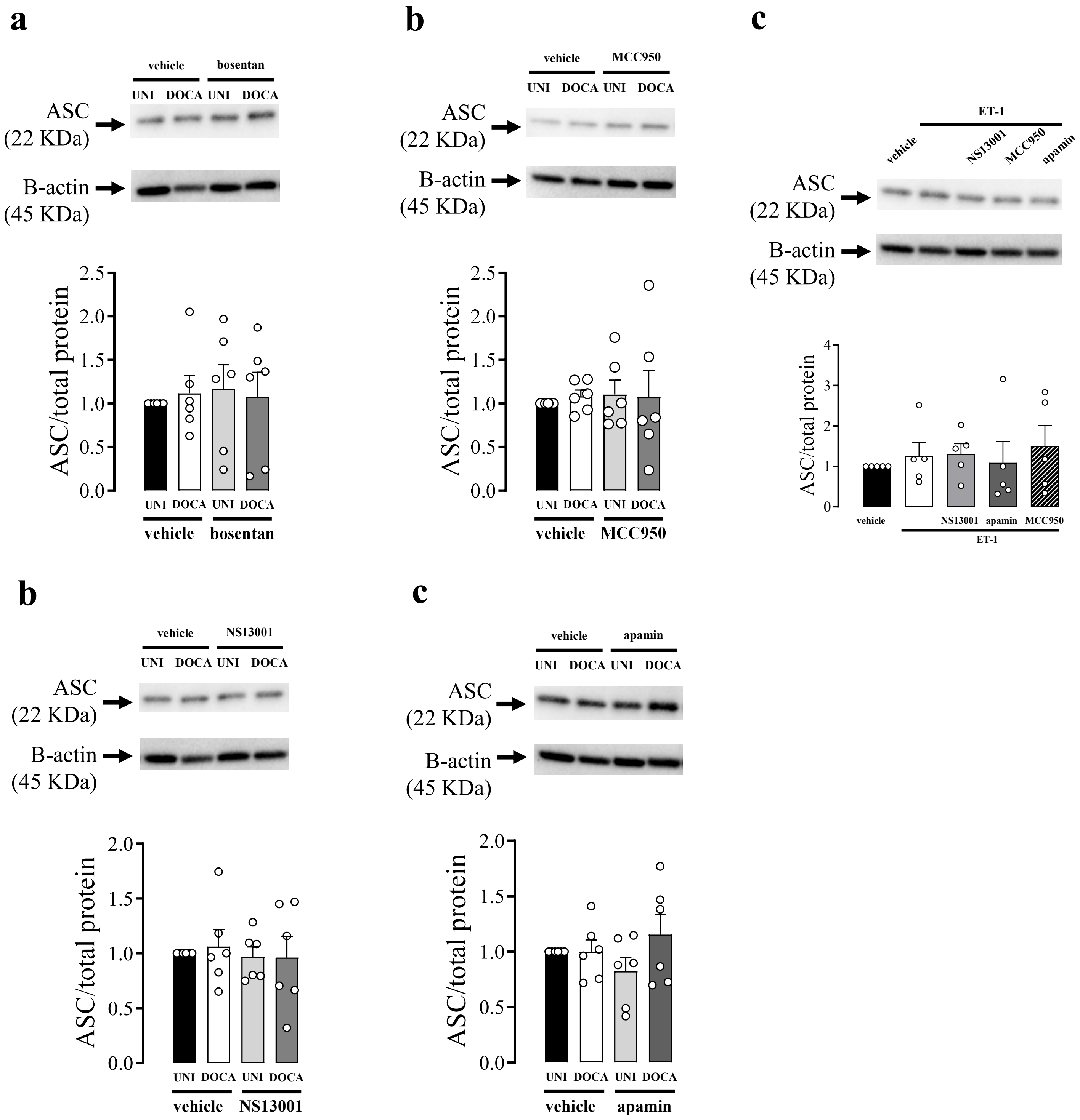

Figure S5. Analysis of the ASC in CC strips of DOCA/salt hypertensive (DOCA) or unilaterally nephrectomized (UNI) rats treated with vehicle, (**a**) bosentan, (**b**) MCC950, (**d**) NS13001, (**e**) apamin. (**c**) ASC expression in CC strips of healthy mice treated with vehicle, or ET-1 in the presence or absence of NS13001, apamin, and MCC950. Data represent the mean ± SEM values of the groups. * $p<0.05$. n=6. Each value was compared by two-way ANOVA followed by The Holm-Šídák post-test. The entire membranes for ASC are shown in Figure S10, S13, S16, and S19.

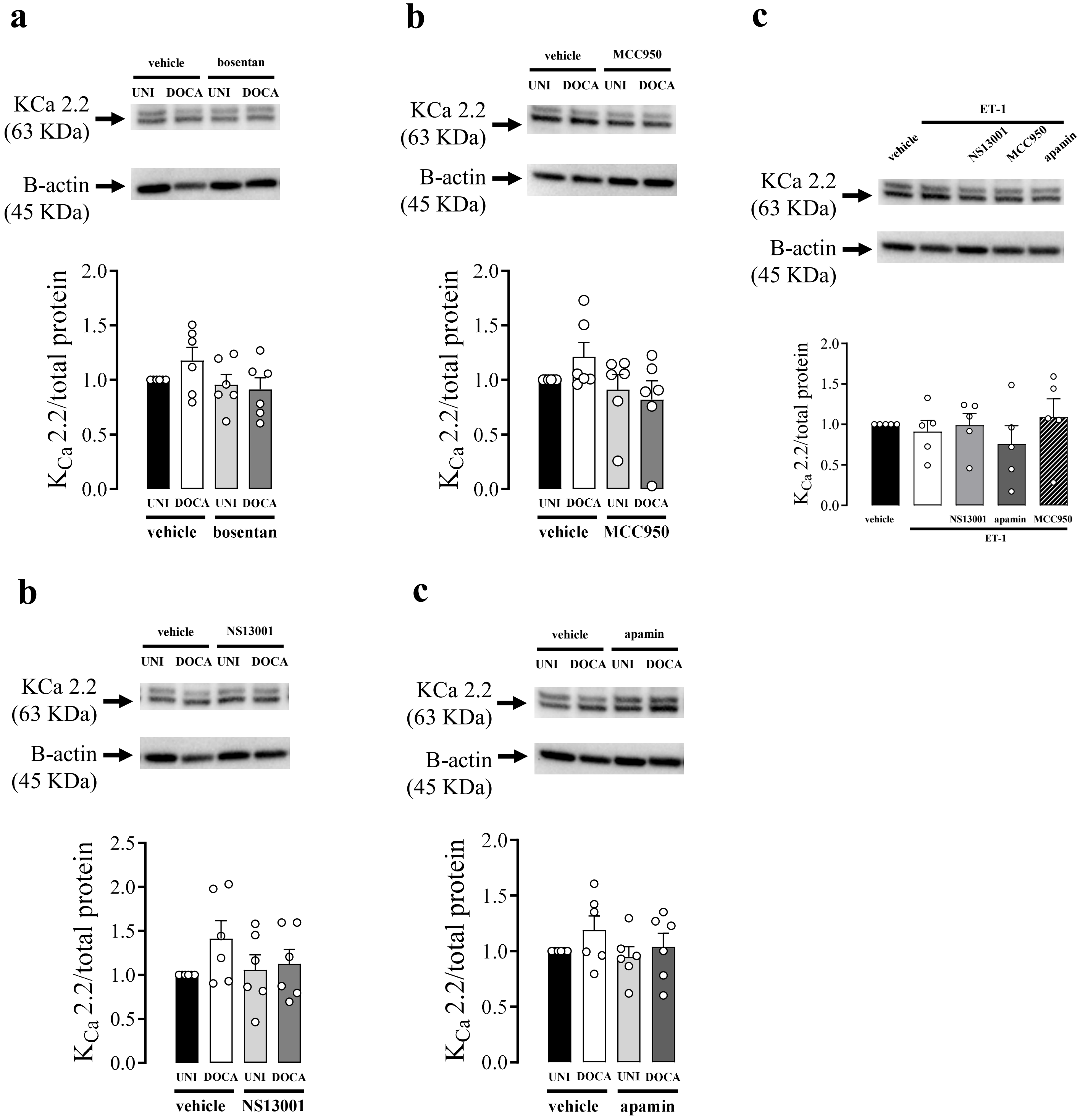

Figure S6. Analysis of the K_{Ca}2.2 in CC strips of DOCA/salt hypertensive (DOCA) or unilaterally nephrectomized (UNI) rats treated with vehicle, (**a**) bosentan, (**b**) MCC950, (**d**) NS13001, (**e**) apamin. (**c**) K_{Ca}2.2 expression in CC strips of healthy mice treated with vehicle, or ET-1 in the presence or absence of NS13001, apamin, and MCC950. Data represent the mean ± SEM values of the groups. * $p<0.05$. n=6. Each value was compared by two-way ANOVA followed by The Holm-Šídák post-test. The entire membranes for ASC are shown in Figure S10, S13, S16, and S19.

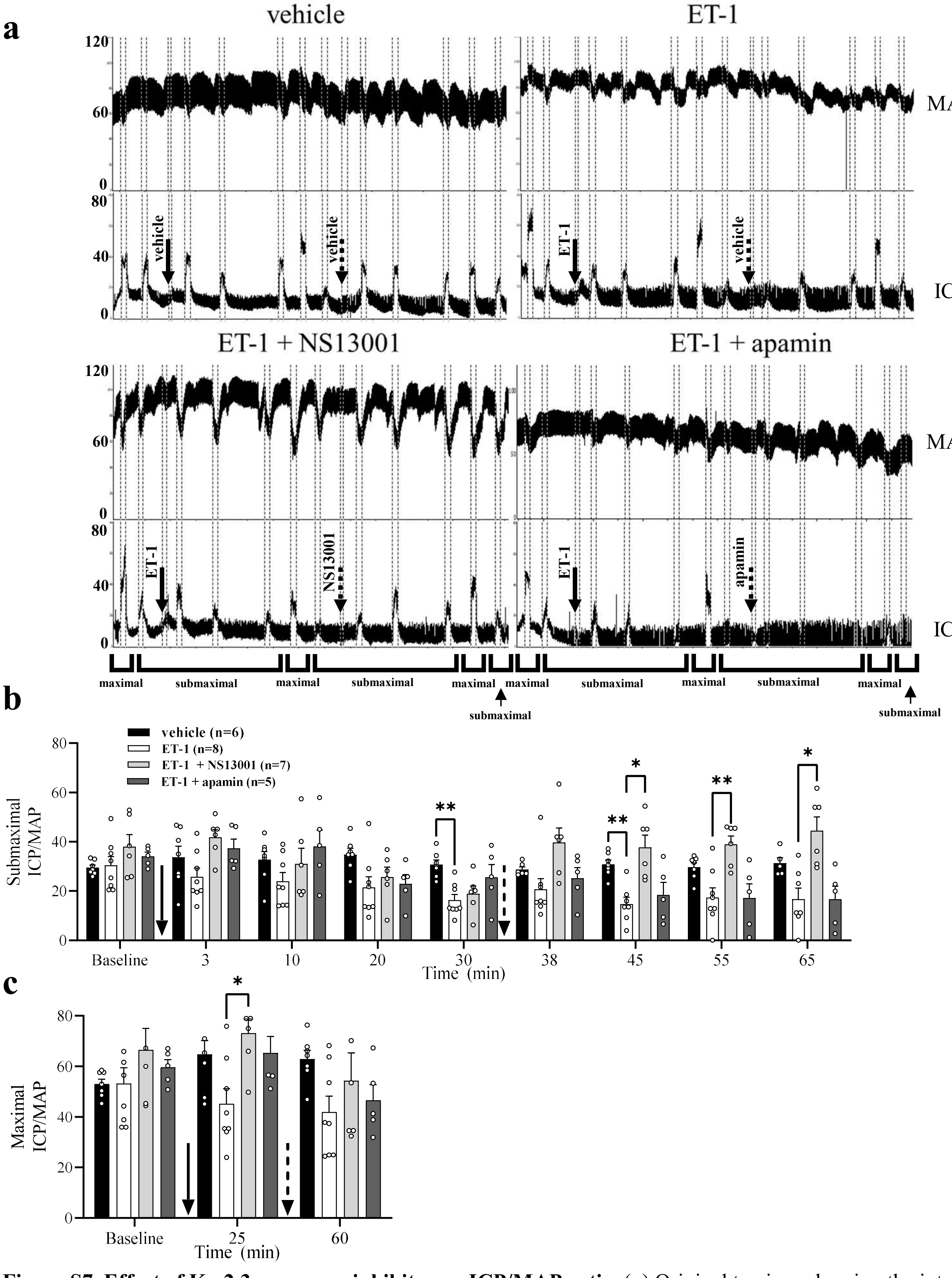

Figure S7. Effect of $K_{Ca}2.3$ opener or inhibitor on ICP/MAP ratio. (**a**) Original tracings showing the intracavernosal pressure (ICP, lower traces) and mean arterial pressure (MAP, upper traces) in response to cavernosal nerve stimulation assessed. The graphics depict the ICP/MAP ratio in response to cavernosal nerve stimulation: (**b**) submaximal (2, 4 or 8 Hz) at 3, 10, 20 and 30 min and (**c**) maximal (16 Hz) at 25 min after the injection of the vehicle followed by a new vehicle administration. Submaximal stimulation at 35, 38, 45, 55, and 65 min and maximal at 60 min, ET-1 followed by vehicle, NS13001 or apamin followed by ET-1 or vehicle. Data represent mean ± SEM of ICP/MAP values. Representative tracings showing changes in intracavernosal pressure and blood pressure in response to electrical stimulation of the cavernosal nerve. *$p < 0.05$. n = 5-9. Each frequency value for the ICP/MAP was performed by repeated-measures two-way ANOVA followed by Holm- Šídák post-test. ICP = intracavernosal pressure; MAP = mean arterial pressure. The continuous arrow represents the first administration at 0 min and the dashed arrow represents the second administration at 35 min

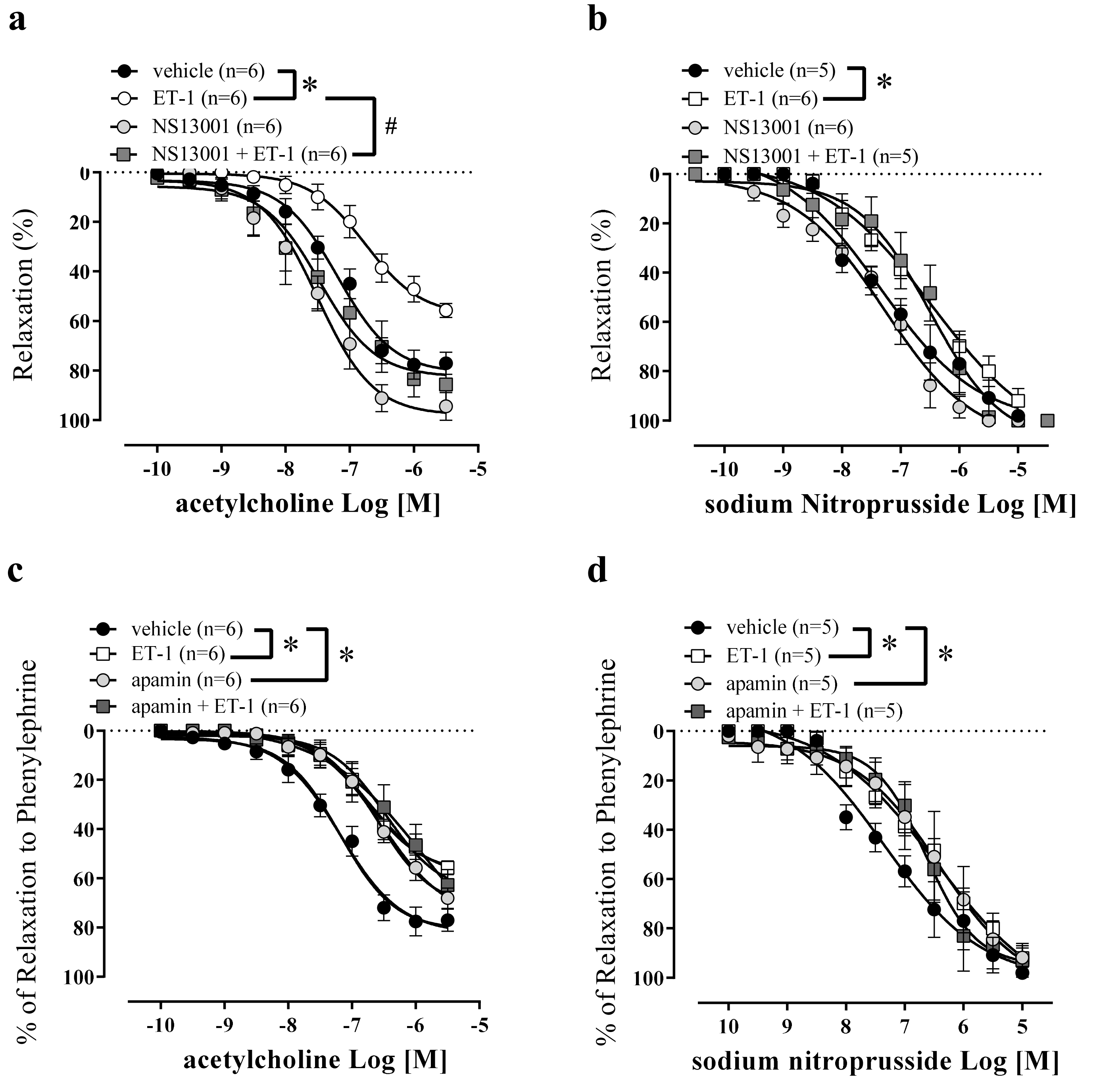

Figure S8. Concentration-response curves to (**a** and **c)**acetylcholine, and sodium (**b** and **d)** nitroprusside in CC strips incubated with (**a** and **b**) NS13001 or (**c** and **d**) apamin of healthy mice. * $p < 0.05$. n = 5-6. The comparison of pEC50 and Emax (table 1) and each value was performed by two-way ANOVA followed by Holm-Šídák post-test.

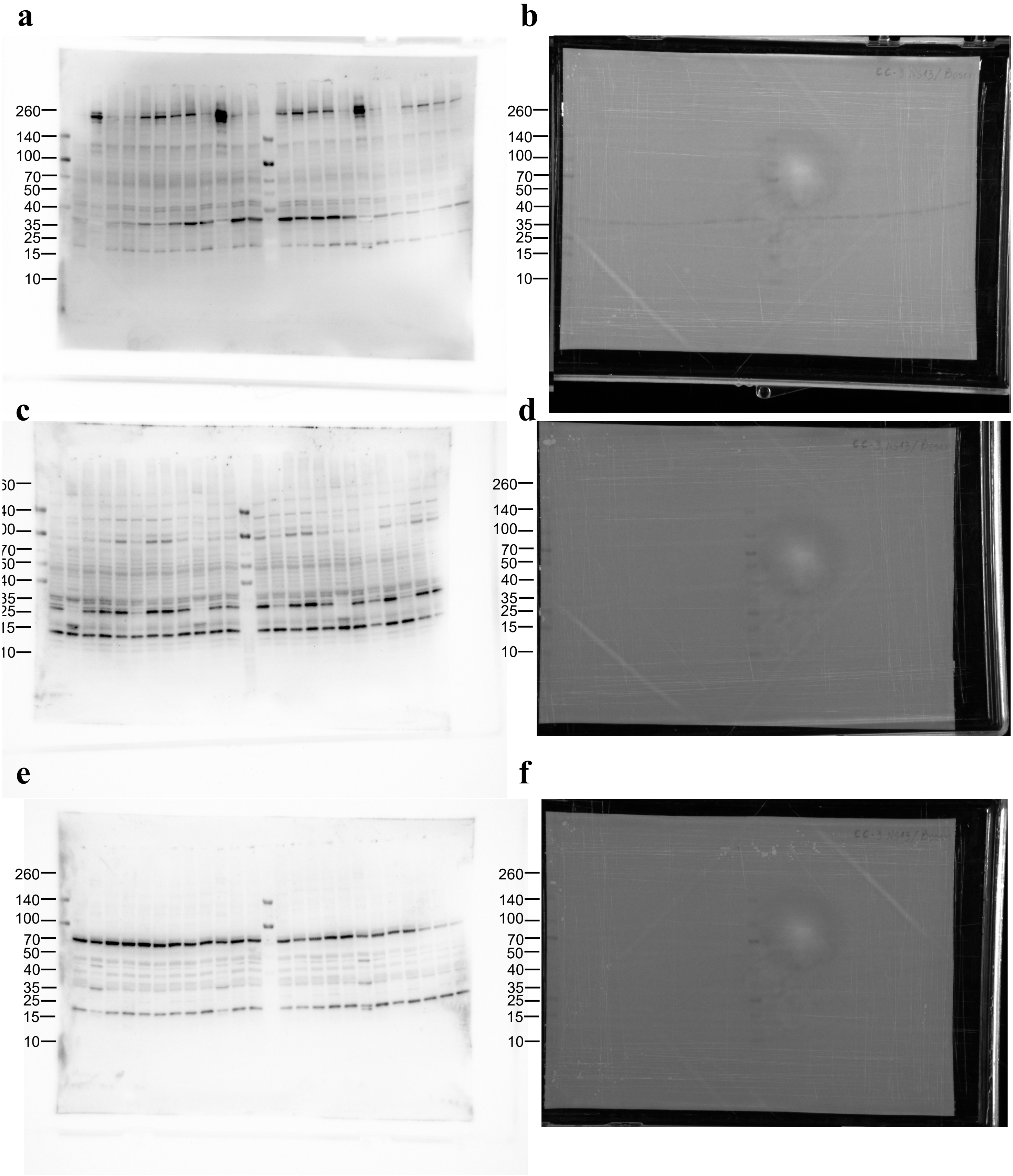

lane 1 – CC UNI vehicle
lane 2 – CC DOCA vehicle
lane 3 – CC UNI bosentan
lane 4 – CC DOCA bosentan
lane 5 – CC UNI vehicle
lane 6 – CC DOCA vehicle
lane 7 – CC UNI bosentan
lane 8 – CC DOCA bosentan

lane 9 – CC UNI vehicle
lane 10 – CC DOCA vehicle
lane 11 – CC UNI bosentan
lane 12 – CC DOCA bosentan
lane 13 – CC UNI vehicle
lane 14 – CC DOCA vehicle
lane 15 – CC UNI NS13001
lane 16 – CC DOCA NS13001

lane 17 – CC UNI vehicle
lane 18 – CC DOCA vehicle
lane 19 – CC UNI NS13001
lane 20 – CC DOCA NS13001
lane 21 – CC UNI vehicle
lane 22 – CC DOCA vehicle
lane 23 – CC UNI NS13001
lane 24 – CC DOCA MCC950

Figure S9. The membranes exposed to cheminoluminescence (**a, c, d**) and epiluminescence (**b, d, f**) to develop the immunoblotting for (**a, b**) pro-caspase-1, caspase-1, (**c, d**) pro-IL-1β, IL-1β, (**e, f**) and K_{Ca} 2.3.

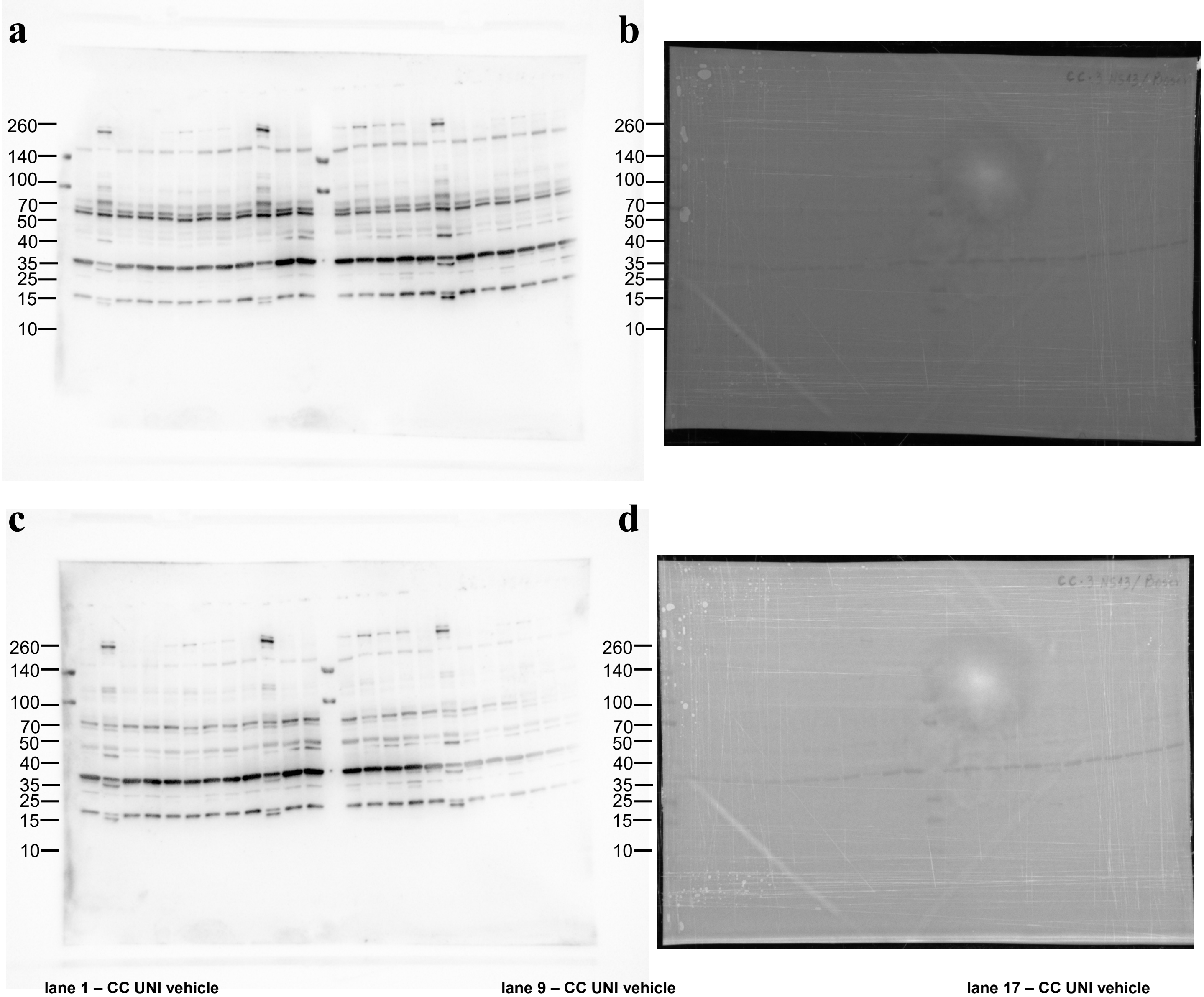

lane 1 – CC UNI vehicle
lane 2 – CC DOCA vehicle
lane 3 – CC UNI bosentan
lane 4 – CC DOCA bosentan
lane 5 – CC UNI vehicle
lane 6 – CC DOCA vehicle
lane 7 – CC UNI bosentan
lane 8 – CC DOCA bosentan
lane 9 – CC UNI vehicle
lane 10 – CC DOCA vehicle
lane 11 – CC UNI bosentan
lane 12 – CC DOCA bosentan
lane 13 – CC UNI vehicle
lane 14 – CC DOCA vehicle
lane 15 – CC UNI NS13001
lane 16 – CC DOCA NS13001
lane 17 – CC UNI vehicle
lane 18 – CC DOCA vehicle
lane 19 – CC UNI NS13001
lane 20 – CC DOCA NS13001
lane 21 – CC UNI vehicle
lane 22 – CC DOCA vehicle
lane 23 – CC UNI NS13001
lane 24 – CC DOCA MCC950

Figure S10. The membranes exposed to cheminoluminescence (**a** and **c**) and epiluminescence (**b** and **d**) to develop the immunoblotting for (**a, b**) K_{Ca} 2.2 and (**c, d**) ASC.

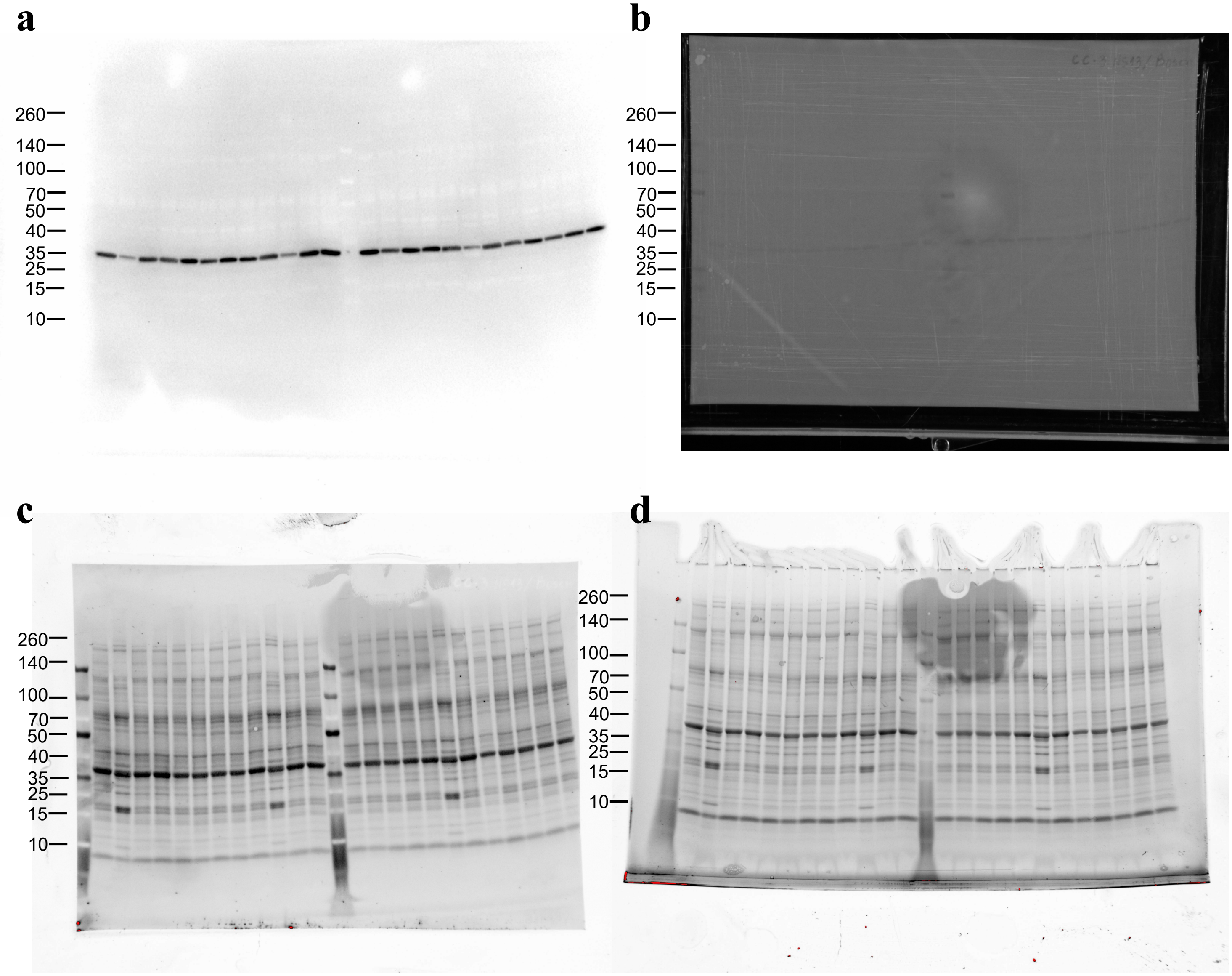

lane 1 – CC UNI vehicle
lane 2 – CC DOCA vehicle
lane 3 – CC UNI bosentan
lane 4 – CC DOCA bosentan
lane 5 – CC UNI vehicle
lane 6 – CC DOCA vehicle
lane 7 – CC UNI bosentan
lane 8 – CC DOCA bosentan
lane 9 – CC UNI vehicle
lane 10 – CC DOCA vehicle
lane 11 – CC UNI bosentan
lane 12 – CC DOCA bosentan
lane 13 – CC UNI vehicle
lane 14 – CC DOCA vehicle
lane 15 – CC UNI NS13001
lane 16 – CC DOCA NS13001
lane 17 – CC UNI vehicle
lane 18 – CC DOCA vehicle
lane 19 – CC UNI NS13001
lane 20 – CC DOCA NS13001
lane 21 – CC UNI vehicle
lane 22 – CC DOCA vehicle
lane 23 – CC UNI NS13001
lane 24 – CC DOCA MCC950

Figure S11. The membranes exposed to cheminoluminescence (**a** and **c**) and epiluminescence (**b** and **d**) to develop the immunoblotting for (**a, b**) β-actin.

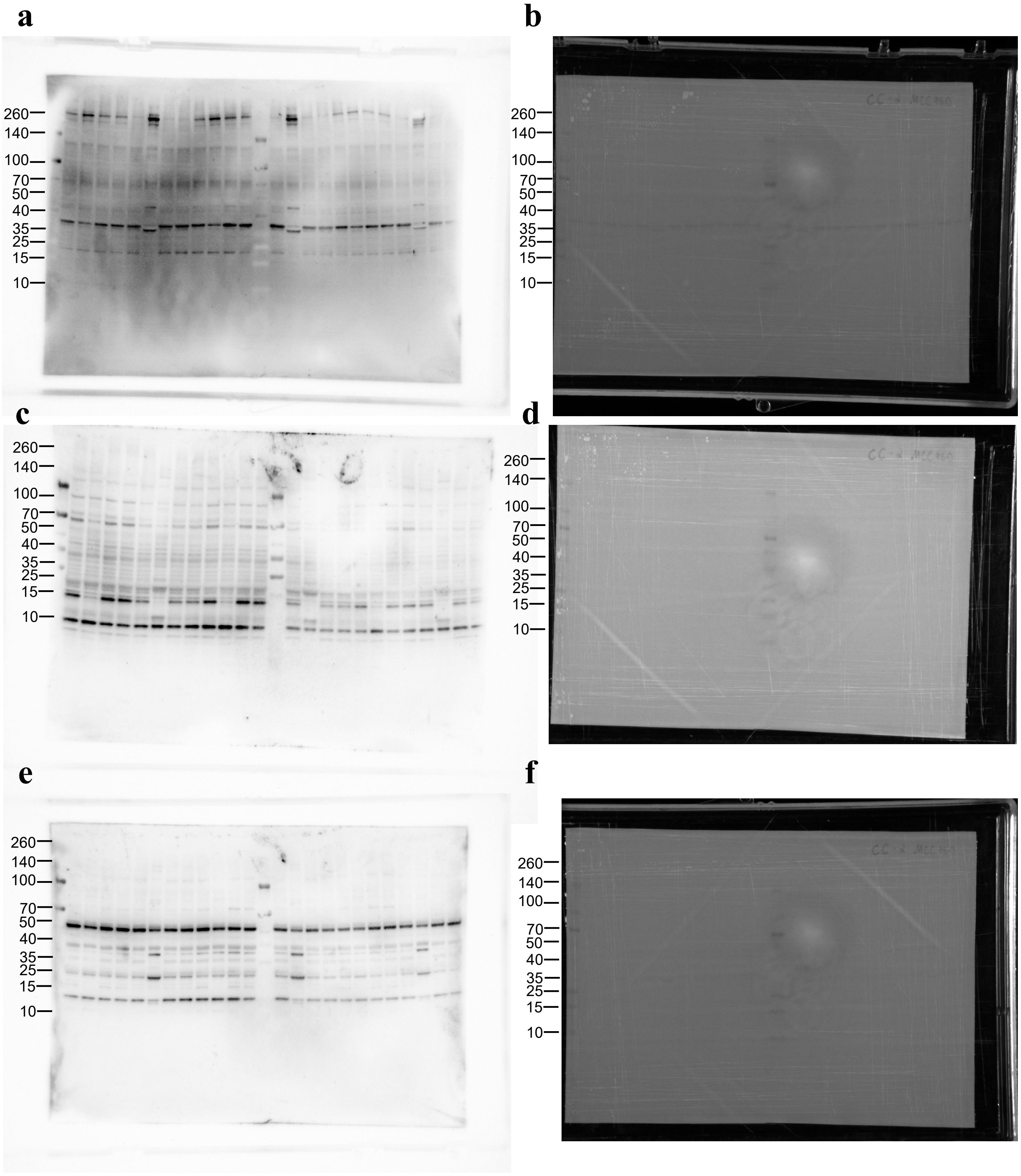

lane 1 – CC UNI vehicle
lane 2 – CC DOCA vehicle
lane 3 – CC UNI MCC950
lane 4 – CC DOCA MCC950
lane 5 – CC UNI vehicle
lane 6 – CC DOCA vehicle
lane 7 – CC UNI MCC950
lane 8 – CC DOCA MCC950
lane 9 – CC UNI vehicle
lane 10 – CC DOCA vehicle
lane 11 – CC UNI MCC950
lane 12 – CC DOCA MCC950
lane 13 – CC UNI vehicle
lane 14 – CC DOCA vehicle
lane 15 – CC UNI MCC950
lane 16 – CC DOCA MCC950
lane 17 – CC UNI vehicle
lane 18 – CC DOCA vehicle
lane 19 – CC UNI MCC950
lane 20 – CC DOCA MCC950
lane 21 – CC UNI vehicle
lane 22 – CC DOCA vehicle
lane 23 – CC UNI MCC950
lane 24 – CC DOCA MCC950

Figure S12. The membranes exposed to cheminoluminescence (**a, c, d**) and epiluminescence (**b, d, f**) to develop the immunoblotting for (**a, b**) pro-caspase-1, caspase-1, (**c, d**) pro-IL-1β, IL-1β, (**e, f**) and K_{Ca} 2.3.

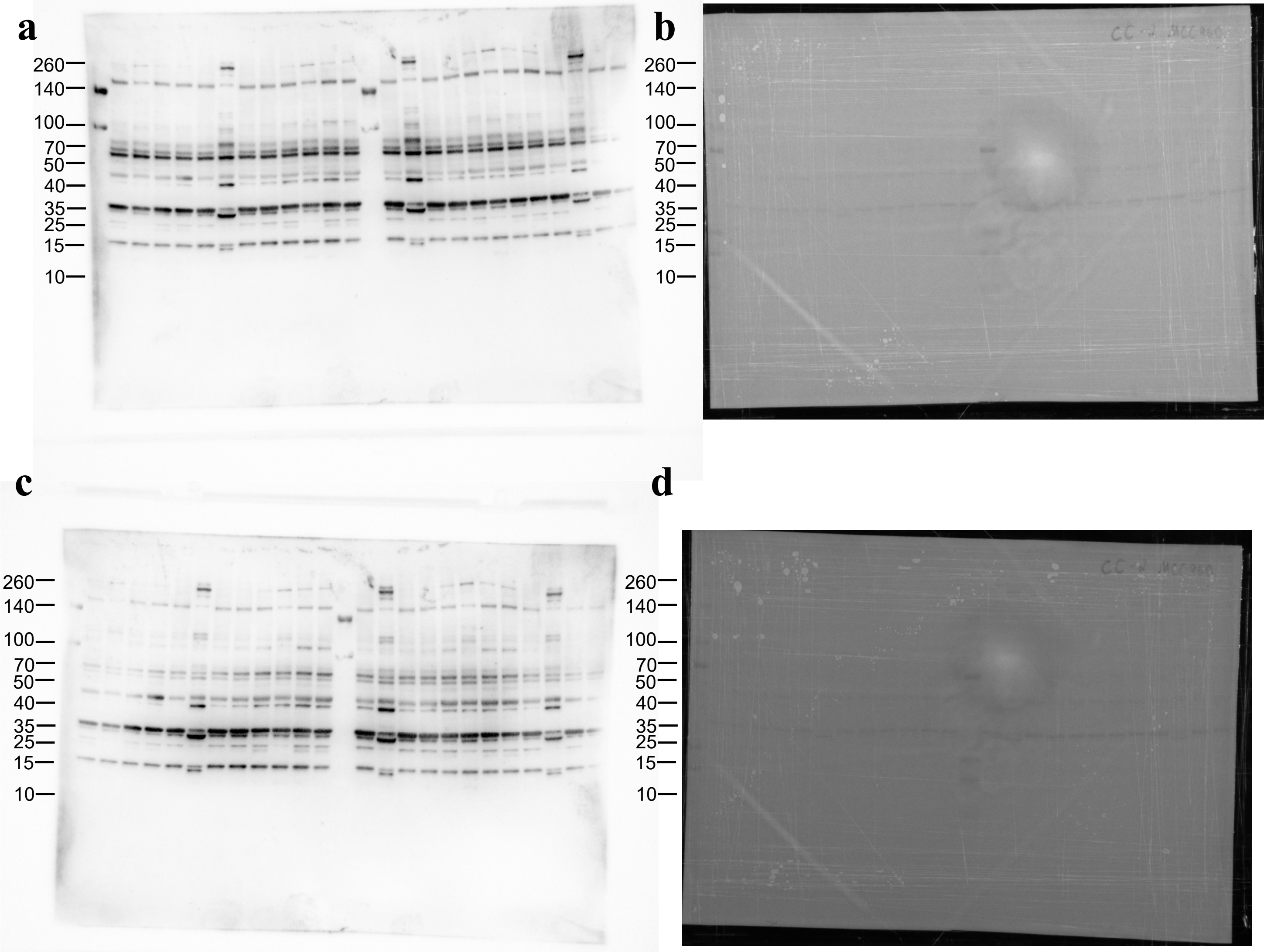

lane 1 – CC UNI vehicle
lane 2 – CC DOCA vehicle
lane 3 – CC UNI MCC950
lane 4 – CC DOCA MCC950
lane 5 – CC UNI vehicle
lane 6 – CC DOCA vehicle
lane 7 – CC UNI MCC950
lane 8 – CC DOCA MCC950
lane 9 – CC UNI vehicle
lane 10 – CC DOCA vehicle
lane 11 – CC UNI MCC950
lane 12 – CC DOCA MCC950
lane 13 – CC UNI vehicle
lane 14 – CC DOCA vehicle
lane 15 – CC UNI MCC950
lane 16 – CC DOCA MCC950
lane 17 – CC UNI vehicle
lane 18 – CC DOCA vehicle
lane 19 – CC UNI MCC950
lane 20 – CC DOCA MCC950
lane 21 – CC UNI vehicle
lane 22 – CC DOCA vehicle
lane 23 – CC UNI MCC950
lane 24 – CC DOCA MCC950

Figure S13. The membranes exposed to cheminoluminescence (**a** and **c**) and epiluminescence (**b** and **d**) to develop the immunoblotting for (**a, b**) K_{Ca} 2.2 and (**c, d**) ASC.

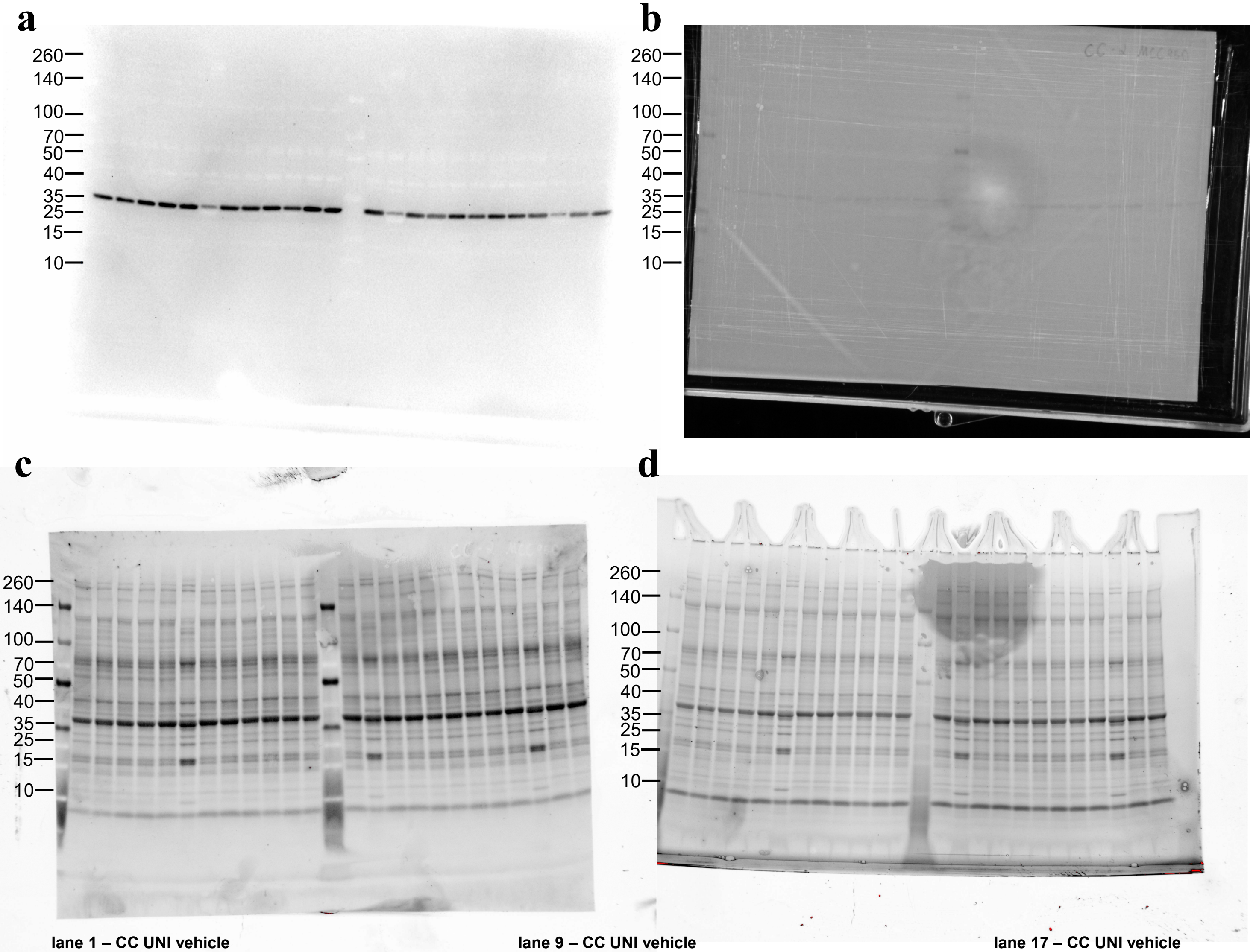

lane 1 – CC UNI vehicle
lane 2 – CC DOCA vehicle
lane 3 – CC UNI MCC950
lane 4 – CC DOCA MCC950
lane 5 – CC UNI vehicle
lane 6 – CC DOCA vehicle
lane 7 – CC UNI MCC950
lane 8 – CC DOCA MCC950

lane 9 – CC UNI vehicle
lane 10 – CC DOCA vehicle
lane 11 – CC UNI MCC950
lane 12 – CC DOCA MCC950
lane 13 – CC UNI vehicle
lane 14 – CC DOCA vehicle
lane 15 – CC UNI MCC950
lane 16 – CC DOCA MCC950

lane 17 – CC UNI vehicle
lane 18 – CC DOCA vehicle
lane 19 – CC UNI MCC950
lane 20 – CC DOCA MCC950
lane 21 – CC UNI vehicle
lane 22 – CC DOCA vehicle
lane 23 – CC UNI MCC950
lane 24 – CC DOCA MCC950

Figure S14. The membranes exposed to cheminoluminescence (**a** and **c**) and epiluminescence (**b** and **d**) to develop the immunoblotting for (**a, b**) β-actin.

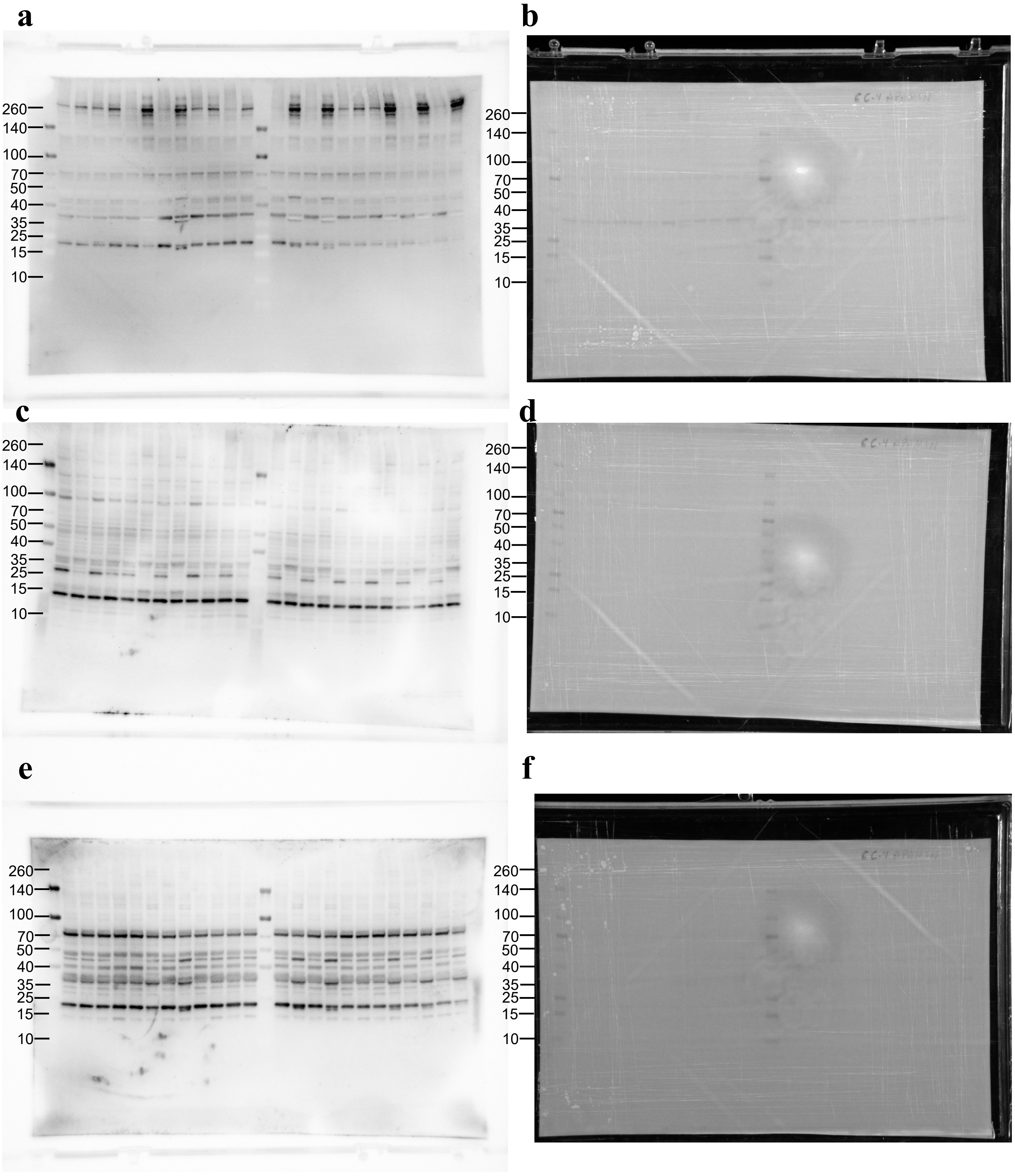

lane 1 – CC UNI vehicle
lane 2 – CC DOCA vehicle
lane 3 – CC UNI apamin
lane 4 – CC DOCA apamin
lane 5 – CC UNI vehicle
lane 6 – CC DOCA vehicle
lane 7 – CC UNI apamin
lane 8 – CC DOCA apamin

lane 9 – CC UNI vehicle
lane 10 – CC DOCA vehicle
lane 11 – CC UNI apamin
lane 12 – CC DOCA apamin
lane 13 – CC UNI vehicle
lane 14 – CC DOCA vehicle
lane 15 – CC UNI apamin
lane 16 – CC DOCA apamin

lane 17 – CC UNI vehicle
lane 18 – CC DOCA vehicle
lane 19 – CC UNI apamin
lane 20 – CC DOCA apamin
lane 21 – CC UNI vehicle
lane 22 – CC DOCA vehicle
lane 23 – CC UNI apamin
lane 24 – CC DOCA apamin

Figure S15. The membranes exposed to cheminoluminescence (**a, c, d**) and epiluminescence (**b, d, f**) to develop the immunoblotting for (**a, b**) pro-caspase-1, caspase-1, (**c, d**) pro-IL-1β, IL-1β, (**e, f**) and K_{Ca} 2.3.

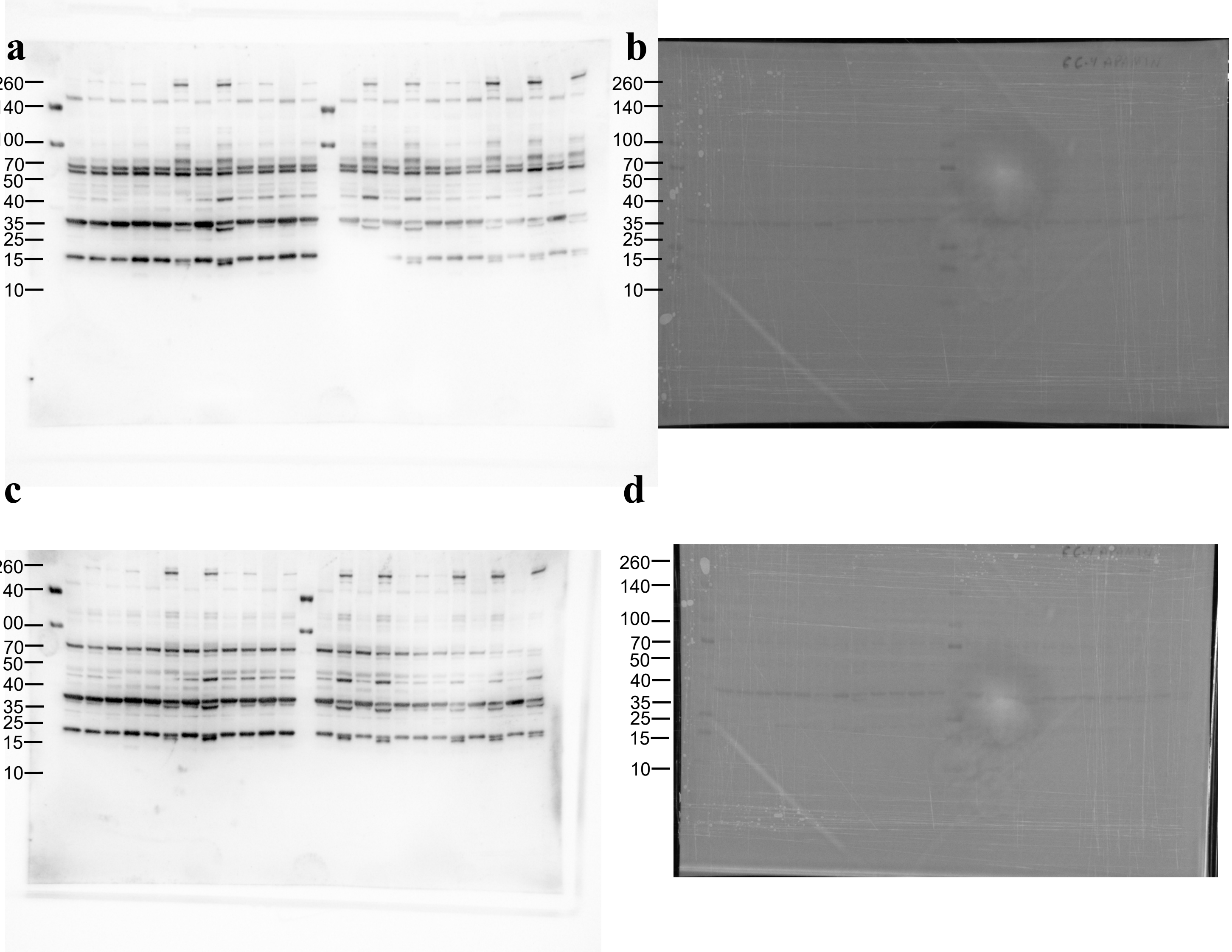

lane 1 – CC UNI vehicle
lane 2 – CC DOCA vehicle
lane 3 – CC UNI apamin
lane 4 – CC DOCA apamin
lane 5 – CC UNI vehicle
lane 6 – CC DOCA vehicle
lane 7 – CC UNI apamin
lane 8 – CC DOCA apamin

lane 9 – CC UNI vehicle
lane 10 – CC DOCA vehicle
lane 11 – CC UNI apamin
lane 12 – CC DOCA apamin
lane 13 – CC UNI vehicle
lane 14 – CC DOCA vehicle
lane 15 – CC UNI apamin
lane 16 – CC DOCA apamin

lane 17 – CC UNI vehicle
lane 18 – CC DOCA vehicle
lane 19 – CC UNI apamin
lane 20 – CC DOCA apamin
lane 21 – CC UNI vehicle
lane 22 – CC DOCA vehicle
lane 23 – CC UNI apamin
lane 24 – CC DOCA apamin

Figure S16. The membranes exposed to cheminoluminescence (**a** and **c**) and epiluminescence (**b** and **d**) to develop the immunoblotting for (**a, b**) K_{Ca} 2.2 and (**c, d**) ASC.

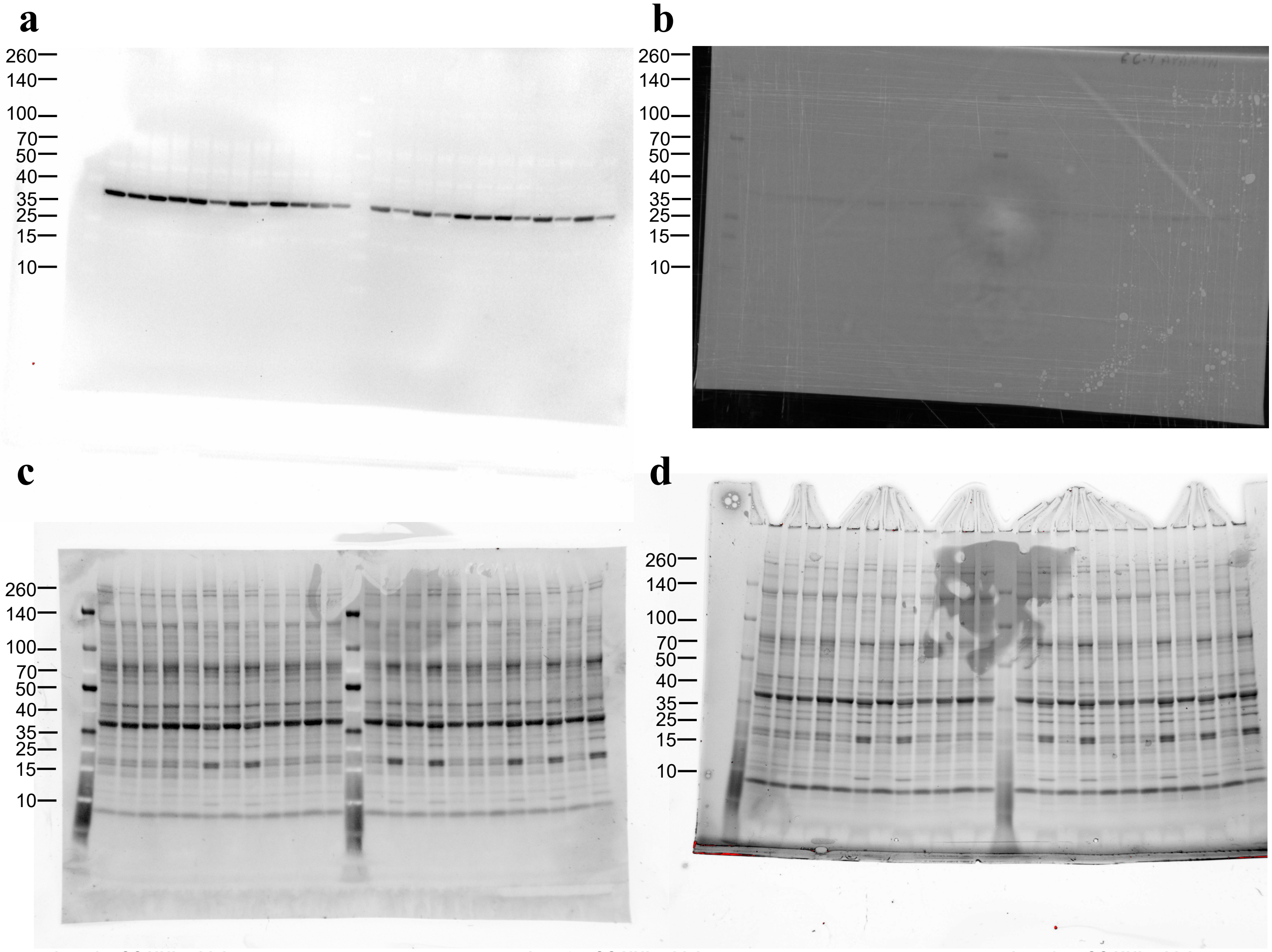

lane 1 – CC UNI vehicle
lane 2 – CC DOCA vehicle
lane 3 – CC UNI apamin
lane 4 – CC DOCA apamin
lane 5 – CC UNI vehicle
lane 6 – CC DOCA vehicle
lane 7 – CC UNI apamin
lane 8 – CC DOCA apamin

lane 9 – CC UNI vehicle
lane 10 – CC DOCA vehicle
lane 11 – CC UNI apamin
lane 12 – CC DOCA apamin
lane 13 – CC UNI vehicle
lane 14 – CC DOCA vehicle
lane 15 – CC UNI apamin
lane 16 – CC DOCA apamin

lane 17 – CC UNI vehicle
lane 18 – CC DOCA vehicle
lane 19 – CC UNI apamin
lane 20 – CC DOCA apamin
lane 21 – CC UNI vehicle
lane 22 – CC DOCA vehicle
lane 23 – CC UNI apamin
lane 24 – CC DOCA apamin

Figure S17. The membranes exposed to cheminoluminescence (**a** and **c**) and epiluminescence (**b** and **d**) to develop the immunoblotting for (**a, b**) β-actin.

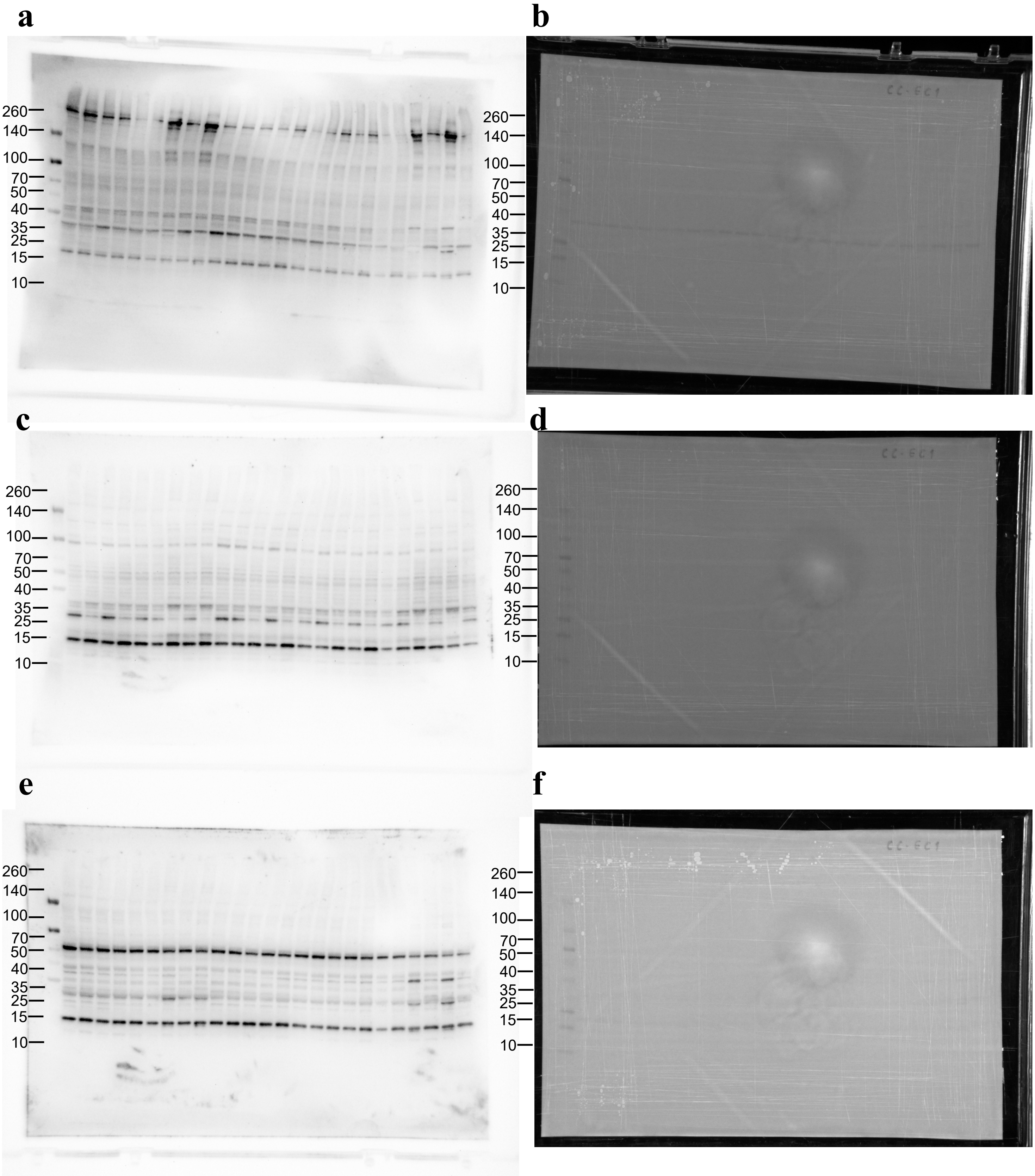

lane 1 – CC vehicle
lane 2 – CC ET-1
lane 3 – CC ET-1 NS13001
lane 4 – CC ET-1 apamin
lane 5 – CC ET-1 MCC950
lane 6 – CC vehicle
lane 7 – CC ET-1
lane 8 – CC ET-1 NS13001
lane 9 – CC ET-1 apamin
lane 10 – CC ET-1 MCC950

lane 11 – CC vehicle
lane 12 – CC ET-1
lane 13 – CC ET-1 NS13001
lane 14 – CC ET-1 apamin
lane 15 – CC ET-1 MCC950
lane 16 – CC vehicle
lane 17 – CC ET-1
lane 18 – CC ET-1 NS13001
lane 19 – CC ET-1 apamin
lane 20 – CC ET-1 MCC950

lane 21 – CC vehicle
lane 22 – CC ET-1
lane 23 – CC ET-1 NS13001
lane 24 – CC ET-1 apamin
lane 25 – CC ET-1 MCC950

Figure S18. The membranes exposed to cheminoluminescence (**a, c, d**) and epiluminescence (**b, d, f**) to develop the immunoblotting for (**a, b**) pro-caspase-1, caspase-1, (**c, d**) pro-IL-1β, IL-1β, (**e, f**) and K_{Ca} 2.3.

c

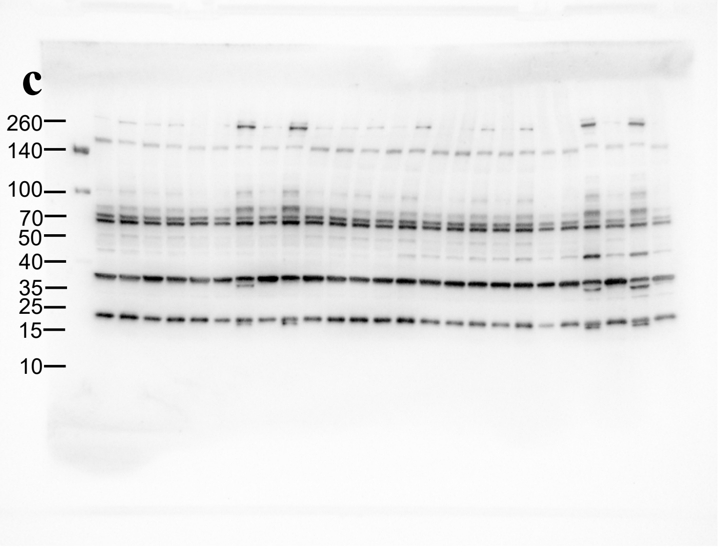

d

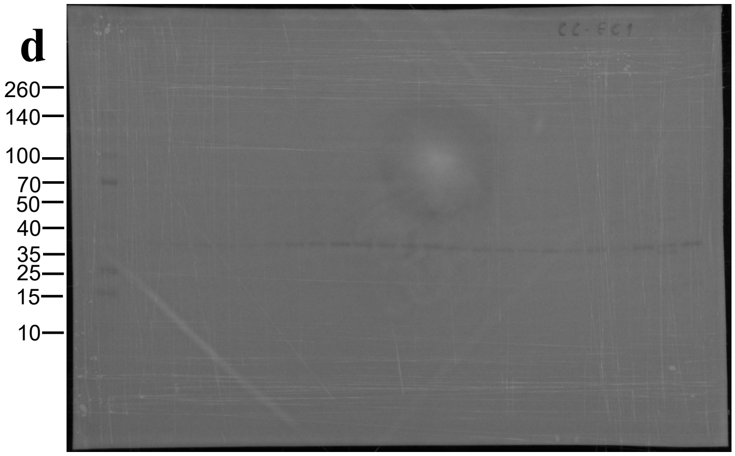

e

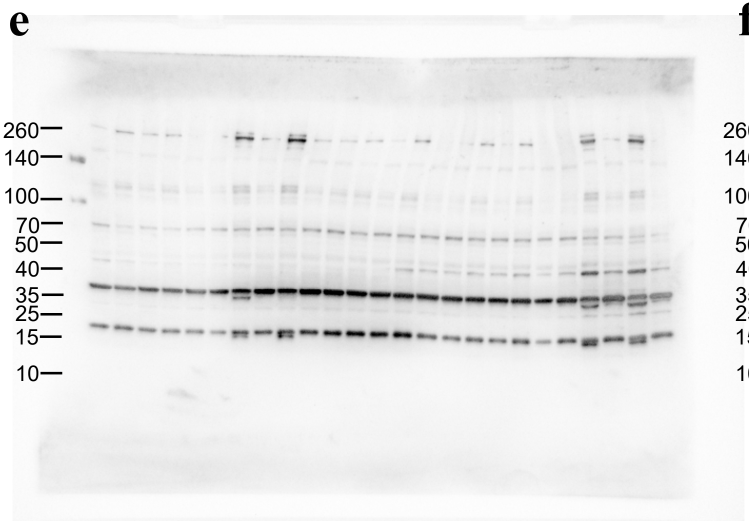

f

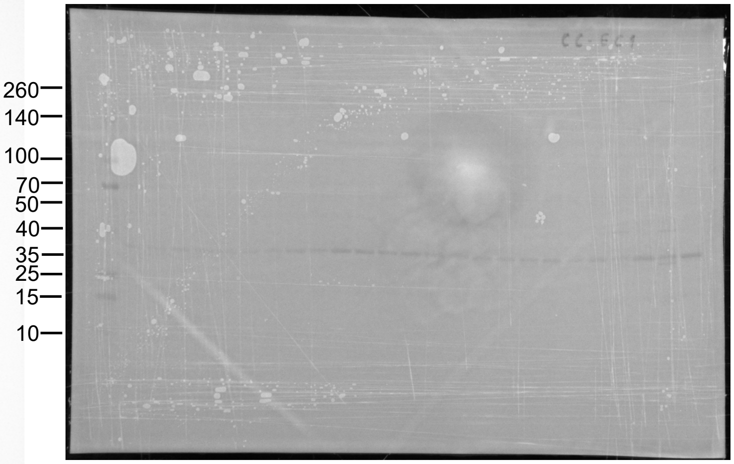

lane 1 – CC vehicle
lane 2 – CC ET-1
lane 3 – CC ET-1 NS13001
lane 4 – CC ET-1 apamin
lane 5 – CC ET-1 MCC950
lane 6 – CC vehicle
lane 7 – CC ET-1
lane 8 – CC ET-1 NS13001
lane 9 – CC ET-1 apamin
lane 10 – CC ET-1 MCC950
lane 11 – CC vehicle
lane 12 – CC ET-1
lane 13 – CC ET-1 NS13001
lane 14 – CC ET-1 apamin
lane 15 – CC ET-1 MCC950
lane 16 – CC vehicle
lane 17 – CC ET-1
lane 18 – CC ET-1 NS13001
lane 19 – CC ET-1 apamin
lane 20 – CC ET-1 MCC950
lane 21 – CC vehicle
lane 22 – CC ET-1
lane 23 – CC ET-1 NS13001
lane 24 – CC ET-1 apamin
lane 25 – CC ET-1 MCC950

Figure S19. The membranes exposed to cheminoluminescence (**a** and **c**) and epiluminescence (**b** and **d**) to develop the immunoblotting for (**a, b**) K_{Ca} 2.2 and (**c, d**) ASC.

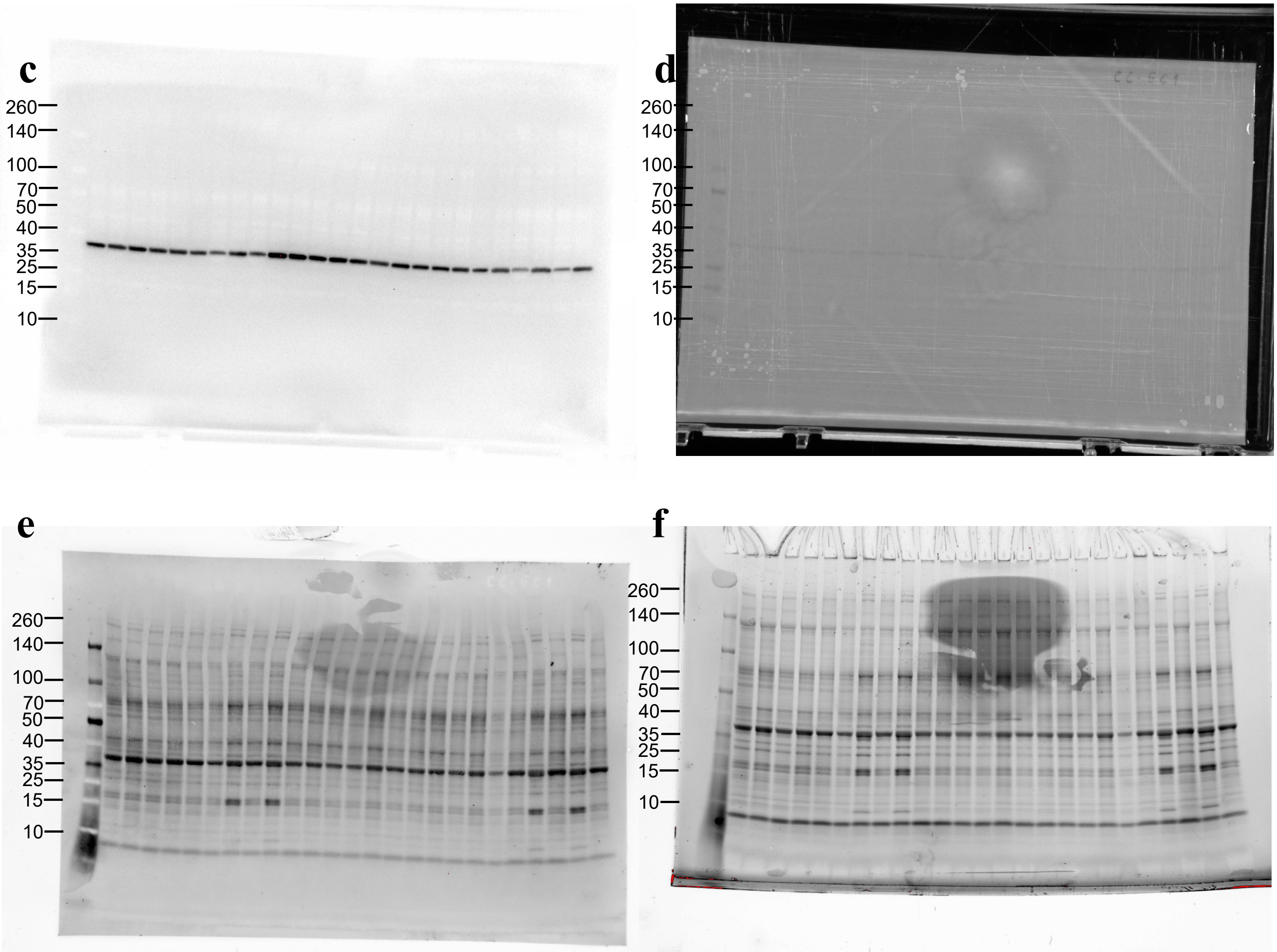

lane 1 – CC vehicle
lane 2 – CC ET-1
lane 3 – CC ET-1 NS13001
lane 4 – CC ET-1 apamin
lane 5 – CC ET-1 MCC950
lane 6 – CC vehicle
lane 7 – CC ET-1
lane 8 – CC ET-1 NS13001
lane 9 – CC ET-1 apamin
lane 10 – CC ET-1 MCC950

lane 11 – CC vehicle
lane 12 – CC ET-1
lane 13 – CC ET-1 NS13001
lane 14 – CC ET-1 apamin
lane 15 – CC ET-1 MCC950
lane 16 – CC vehicle
lane 17 – CC ET-1
lane 18 – CC ET-1 NS13001
lane 19 – CC ET-1 apamin
lane 20 – CC ET-1 MCC950

lane 21 – CC vehicle
lane 22 – CC ET-1
lane 23 – CC ET-1 NS13001
lane 24 – CC ET-1 apamin
lane 25 – CC ET-1 MCC950

Figure S20. The membranes exposed to cheminoluminescence (**a** and **c**) and epiluminescence (**b** and **d**) to develop the immunoblotting for (**a, b**) β-actin.
